# Abdominal Surgery and Listerism

**Published:** 1881-11-20

**Authors:** 


### ABDOMINAL SURGERY AND LISTERISM.

---

The three topics of interest in the Surgical section of the International Medical Congress were Abdominal Surgery, "Intra Peritoneal," the programme had it, Modern Lithoirity,—they wont say "Lytholapaxy" over here,—and the treatment of Wounds to secure Union by First Intention.

I may say parenthetically that the mode of procedure was for some one, or more, who had previously promised it, to read a paper upon the subject, and the discussion of those papers was taken up by the gentlemen appointed for that duty, whose names were printed upon the programme, and who were called in regular order by the president. And I may also say, right here, that every delegate was anxious to ascertain the exact position of "Listerism" in the convention. It was noticeable that early in the sessions when certain men, who shall be nameless, *seemed* to try to test the matter by initiating applause at every allusion to anticeptic surgery there was very little response. Mr. Lister himself was always and everywhere heartily received. But it required no great sagacity to see that the majority of surgeons were *reserved* in the matter. But more of this further on.

Spencer Wells read a paper. He took strong Listerian ground, and said that now he had given up drainage altogether, so great was his faith in antiseptic surgery. Several others, Volkmann especially, followed in a similar strain. Then Marion Sims arose, and while he declared for Listerism he advocated drainage, and reminded Mr. Wells of a case (ovariotomy), in which he assisted him in a bad operation,—bad on account of adhesions,—and the patient *almost* died, but at last nature opened the abdominal wound and discharged a large amount of fetid fluid, and immediately she recovered. Finally came Mr. Keith o close the discussion. Never in the history of surgery did a few modest words make such a record in the "currents of expectant thought" as his.

It has been said, and was repeated by Volkmann and Kuget, in this discussion, that intra-peritoneal surgery was the "touchstone of Listerism." Professor Keith hss been quoted the world over, again and again, as not only a warm disciple of Lister, but as illustrating in his remarkable success in ovariotomy, *more than any other surgeon*, the value of the anticeptic, or rather, the Listerian method. No one can deny this.

So slowly were his few words uttered that I can almost repeat every one *verbatim*.

You can imagine the effect much better than I can describe it when he said that for several months past he had "abandoned the anticeptic treatment altogether." "True," he said, "I had eighty successive recoveries under Lister's method, and *stopping there* it would be a wonderful showing. *But out of the next twenty-five I lost seven.* One died of acute septicæmia, in spite of the most thorough anticeptic precaution; three of "unquestionable carbolic acid poisoning; one of renal hæmorrhage." He went on to say that out of the eighty consecutive cases (or rather he said it first) many came too near dying; that a large number got a high temperature—105°, 106°, 107° Fahrenheit—the evening following the operation, but he said, "they happened to pull through." He then said that since he had for four months past abandoned the anticeptic method and relied upon perfect cleanliness, care in controlling hæmorrhage, and thorough drainage, his cases were giving him much less trouble, and he was getting more satisfactory results.

He now stopped for a few moments; hesitating, as he must have realized the importance of his words, knowing that the whole world—surgical—was lending a "listening ear" to his utterance. The silence was

"audible." Then he raised his head, and looking his audience squarely in the face, he said, "Gentlemen, I have felt it my duty to make these statements, for *they are true*," and took his seat. I shall not attempt to describe the applause, nor the effect of his statements. Professor Keith, by the way, told me privately that he almost died himself from using the carbolic acid so much. He got renal hæmorrhage and debility to an alarming degree. He said, morever, that he never had great faith in it, and should not have continued its use so long—I mean the "Lister method"—but for the fact that so many eminent men were carried away with it; and if, after his remarkable series of cases, he had changed, and lost seven out of twenty-five, as he did, without Listerism, all the world—he himself—would have attributed the result to the change.

One thing is certain; Mr. Keith's statements, in conneetion with those of others *and his own experience*, put Mr. Lister in a very unpleasant position; for he was put down on the programme to close the discussion on the treatment of wounds to secure union by first intention, which took place on Monday, 8th inst. Although four days had elapsed, he had no answer. To show how deeply he was impressed by all that had been said, he began his remarks, which were extemporaneous instead of written, as was expected, by saying that he never had admitted that abdominal surgery was the "touchstone of Listerism," and to the surprise and dismay of his followers went on to argue that, with the rapidity with which wounds of the peritoneum heal and the remarkable absorbing power of that membrane, and therefore its ability to take care of its exudates, he "doubted very much" whether, in the hands of a skillful, careful operator, it was not better to dispense with the antiseptic plan. I realize how important are the statements I am making, and lest some of your readers may think that they are open to criticism as to accuracy, I will say that I sat near enough to hear every syllable uttered, and I pledge my honor as a man and surgeon for the absolute accuracy of every statement, though I took a few notes.

Then, seeming to realize the danger of administering such wonderful absorbent qualities to the peritonæum, he went on to say that he had recently made some experiments that surprised him very much, which proved that serum or bloody serum was "a very poor soil for the development of germs from contact with air-dust, and that blood clots were still more sterile. Indeed, it was very difficult to make them grow or develop at all, unless diluted with water." By the way, he declared that he had witnessed free cell development in a blood clot.

And these remarkable facts, said he, "at once call in question the necessity of the spray."

He then went on to say that he was not yet ready to give up the spray, but if simple irrigation or lavation should prove as good, he would say, "*Fort mit dem spray;*" and he further said, "I am not at all sure but that before the next meeting, two years hence, I shall have abandoned the spray altogether." (His recent house surgeon says that he has lost all confidence in its utility.)

As to carbolic acid, he said, "I am forced to admit its unfortunate character." That was all; not a word about oil of eucalyptus or any other substitute. He kept referring again and again to abdominal surgery, but his manner showed to everybody that he was upset.

He gave no statistics, no large comparisons, as was expected by his disciples. He referred to the excellent results in two cases of recent operation, saying that "I could hardly believe I should have got such results without the antiseptic plan; I did not before I used it."

And this is the fault that the best surgeons here find with him. They are all ready and glad to give him or any other man credit for all he has really done, and they all admit that Mr. Lister has done much to improve surgery, especially German surgery. I need not explain. But they very properly say, "With his unprecedented opportunities, both in his host of followers, why don't he give us large and complete statistics? Instead, he only gives either isolated cases or small group of successful ones, such as may be found under almost any plan." I quote one of London's most eminent and fair-minded men.

It was curious to watch the effect of the thing. I have alluded to the impression produced by Keith's remarks. As Lister was speaking, one of his ardent admirers—I mean an admirer of his mode of dressing; I am not discussing the man, who is an earnest, hard-working, accomplished gentleman—turned to me, and said, "I would never have believed Professor Lister would have admitted that." Another said, "Well, if Lister abandons the spray and carbolic acid, giving us no substitute, where is 'Listerism?' We had drainage, we had animal ligatures, we had air-proof dressings, before." And so on. Every little group of surgeons was discussing the matter; those who had never accepted the Listerian method being quite as much surprised as its warmest adherents.

"Mein Gott!" said a German whom I did not know, "Lishtereism ist todt." "Fort dem Spray? Fort dem Acid Carbolique? Was gibts zu bleiben?"—*Boston Medical Journal.*

